# Hypertension in Pregnancy and Preeclampsia: Variation in Clinical Management Strategies Among Obstetric/Gynecologic Provider Type

**DOI:** 10.1089/whr.2023.0031

**Published:** 2023-10-25

**Authors:** Sharla Smith, Patrick Kim, Sharon Fitzgerald Wolff, Megha Ramaswamy, Tracie Collins

**Affiliations:** ^1^ Department of Population Health, University of Kansas Medical Center, Kansas City, Kansas, USA.; ^2^ School of Medicine, University of Kansas Medical Center, Kansas City, Kansas, USA.; ^3^ College of Population Health, University of New Mexico, Albuquerque, New Mexico, USA.

**Keywords:** hypertension, aspirin, maternal and fetal medicine, health disparities

## Abstract

**Background:**

Preeclampsia, a condition in pregnancy characterized by new onset high blood pressure and proteinuria, complicates 2%–8% of pregnancies globally. Early detection, careful monitoring, and treatment of high blood pressure are crucial in preventing mortality related to preeclampsia disorders. There is limited data that examines obstetric/gynecologic (OBGYN) provider-type practices concerning management of hypertensive disorders of pregnancy to reduce early onset preeclampsia (EOP). We assessed the knowledge and practice patterns of OBGYN management to reduce EOP.

**Methods:**

We conducted a semistructured survey with OBGYN residents, maternal-fetal medicine fellows, and attending physicians (OBGYN and family medicine) at a single academic medical center to assess the management of hypertensive disorders to EOP.

**Results:**

Thirty-one participants (71% residents/fellows 29% attendings) completed the survey. Seventy-eight percent of attendings indicated they discuss blood pressure and preeclampsia with all patients compared to 50% of residents/fellows (*p* = 0.31). Eighty-nine percent of attendings reported they are extremely likely to monitor high-risk patients compared to 36% of residents/fellows (*p* = 0.07).

**Conclusion:**

Attending physicians were more likely to appropriately manage hypertension in women at risk for pregnancy compared to residents/fellows. Further research is needed on monitoring high-risk patients.

## Introduction

Hypertensive diseases of pregnancy are serious maternal morbidities, occurring in 6%–8% of all pregnancies.^1^ Gestational hypertension and preeclampsia are risk factors for other forms of maternal morbidities (as well as neonatal morbidity and mortality), making these diseases of reproductive-aged women a particularly important public health problem. Women with gestational hypertension risk further progression to severe hypertension, preeclampsia, or eclampsia. Furthermore, women with preeclampsia are predisposed to convulsions, abruptio placentae, disseminated intravascular coagulation, cerebral hemorrhage, pulmonary edema, renal failure, and liver hemorrhage.^2^ The risks posed by preeclampsia to the fetus include severe growth retardation, hypoxemia, acidosis, premature birth, and death.^3^ Early onset preeclampsia (EOP) is defined as preeclampsia which develops before 34 weeks' gestation, whereas late-onset preeclampsia (LOP) develops after 34 weeks' gestation. Although EOP occurs less frequently than LOP, African American and hypertensive patients are at greater risk to develop EOP over LOP.^4^ Because preeclampsia poses serious health risks to these women and fetuses, preventing the development of EOP in high-risk patients is a high-priority goal for obstetric providers. Aspirin is the leading candidate and drug of choice for reducing the risk of developing EOP, but optimal dosing and timing are still under investigated.

Current data from randomized controlled trials and meta-analyses suggest aspirin reduces the risk of preterm pre-eclampsia in high-risk patients if initiated before 16 weeks of gestational age, and the daily dose is greater than 100 mg, up to 150 mg.^5–7^ However, variations in organizational guidelines for prescribing aspirin still exist. The American College of Obstetricians and Gynecologists (ACOG) and the United States Preventive Services Task Force (USPSTF) both recommend 81 mg/day after 12 weeks' gestation and ACOG specifies an optimal start date before 16 weeks. The World Health Organization (WHO) recommends 75 mg/day before the 20-week mark. The International Federation of Gynecology and Obstetrics (FIGO) recommends 150 mg/day starting at 11–14 weeks gestation. Therefore, due to wide variations in available guidelines, high-risk patients typically follow aspirin treatment plans largely determined by their obstetric providers' preferences. There is limited data on aspirin prescription practices about preeclampsia, but one study suggests that differences in patient management exists across different provider training levels.^8^ The results demonstrated that maternal fetal medicine (MFM) fellowship-trained attending physicians were more likely than residents, fellows, and advanced care providers to prescribe aspirin to patients with chronic hypertension, whereas residents and fellows were more likely to prescribe it for diabetic patients. In addition, the study found discrepancies were present between current management guidelines and provider practice.

There is limited data specific to obstetric/gynecologic (OBGYN) aspirin prescription patterns or barriers and facilitators to providing obstetric care for patients at high risk for EOP. The purpose of this study is to describe and compare clinical management strategies and aspirin prescribing patterns for patients at risk for preeclampsia between providers at various training levels (attending physician, MFM fellow, resident physician) in OBGYN and Family Medicine.

## Methods

### Participants and setting

We conducted a single-center, cross-sectional study at a large Midwest academic medical center from April to September 2021. Physicians from the Departments of Obstetrics and Gynecology and Family Medicine were invited to complete an online survey that consisted of questions centered on the management of hypertension and EOP. Physicians were provided with a link to complete the survey during faculty meetings and *via* a recruitment e-mail sent from department administrators.

This project was reviewed and approved by the University of Kansas School of Medicine Institutional Review Board. All participants provided written informed consent before survey completion. All study information was stored in a secure Research Electronic Data Capture database hosted by the University of Kansas Medical Center.^9^

### Measures

Demographic data collected included age, gender, race/ethnicity, and training level (resident, MFM fellow, attending). Practice preferences regarding the management of hypertension and EOP were asked, including information about screening and monitoring for hypertension and EOP, aspirin prescribing practices, and barriers and facilitators to providing obstetric care related to hypertension. Physicians were classified into two groups: trainees (including residents and MFM fellows) and attending physicians.

### Data analysis

Descriptive statistics were assessed with frequencies/percent. Differences in treatment recommendations by training level were compared using Fisher's exact tests. Significance was set *a priori* at *α* = 0.05. All analysis were conducted using SAS, Version 9.4. (SAS Institute, Inc., Cary, NC).

## Results

The survey response rate was 57%. Among those completing the survey, 71% were residents/fellows and 29% were attending physicians. Sixty eight percent of participants reported that their age was between 25 and 34 years. Most participants reported being of white race (74%) (Table 1).

**Table 1.** Participant Characteristics

	Total (***n*** = 31)	Resident/fellow (***n*** = 22)	Attending (***n*** = 9)
Age (years), *n* (%)			
25–34	21 (68)	20 (90)	1 (11)
35–44	6 (26)	2 (10)	6 (67)
45–55	2 (7)	0	2 (22)
Race, *n* (%)			
White	23 (74)	16 (73)	7 (78)
Hispanic/Latinx	2 (6)	1 (5)	1 (11)
Black	4 (13)	4 (18)	0
Native American/Pacific Islander	2 (6)	1 (5)	1 (11)

### Screening and monitoring for hypertension and EOP

About 58% of physicians (residents/fellows, and attendings) reported that they discuss hypertension with all pregnant patients. Although not statistically significant, there were differences in discussion of hypertension between training levels, with 78% of attendings, 50% of residents/fellows reporting that they discuss hypertension with all patients (*p* = 0.31).

Nearly 90% of attending physicians reported that they would be extremely likely to provide additional monitoring for high-risk patients for hypertension and preeclampsia, compared to 36% of residents (*p* = 0.07). The most reported recommendation for additional monitoring included increased monitoring of vitals and lab work (84%), followed by recommendations for patients to check their blood pressure daily at home (74%). Other reported recommendations were increased frequency of physician visits (61%), referrals to high-risk obstetricians (58%), and counseling the patient (65%). Less than half of surveyed physicians (residents/fellows and attendings) reported that they would recommend following a risk factor checklist (48%).

All physicians reported that they would prescribe low-dose aspirin to manage hypertension or preeclampsia. Additional recommendations for management of hypertension reported by physicians included providing patient education (94%), recommending lifestyle modifications (77%), and increasing blood pressure monitoring and ultrasound frequency (68%). About 10% of physicians (residents/fellows and attendings) reported they would prescribe magnesium sulfate, which was differed to by provider type (22% attending physicians, 5% residents/fellows (Table 2).

**Table 2.** Screening and Monitoring for Hypertension and Early Onset Preeclampsia

	Total (***n*** = 31)	Resident/fellow (***n*** = 22)	Attending (***n*** = 9)	***p***
Discusses blood pressure/hypertension with all patients, *n* (%)	18 (58)	11 (50)	7 (78)	0.3075
Provides educational material about high blood pressure/hypertension to all pregnant women, *n* (%)	4 (13)	2 (9)	2 (22)	0.6541
Likelihood of monitoring high risk-patients, *n* (%)				0.0665
Somewhat unlikely	1 (3)	1 (5)	0	
Neutral	3 (10)	3 (14)	0	
Somewhat likely	11 (35)	10 (45)	1 (11)	
Extremely likely	16 (52)	8 (36)	8 (89)	
Recommendations for additional monitoring, *n* (%)				
Risk factor checklist	15 (48)	10 (45)	5 (56)	0.9076
Vitals and lab work	26 (84)	18 (82)	8 (89)	>0.99
Counseling	20 (65)	14 (64)	6 (67)	>0.99
Daily self-blood pressure checks	23 (74)	17 (77)	6 (67)	0.8488
Referral to high-risk obstetrician	18 (58)	13 (59)	5 (56)	>0.99
Increased frequency of physician visits	19 (61)	12 (54)	7 (78)	0.4291
Recommendations for management, *n* (%)				
Prescribe low-dose aspirin	31 (100)	22 (100)	9 (100)	1.0
Prescribe magnesium sulfate	3 (10)	1 (5)	2 (22)	0.3898
Increased blood pressure and ultrasound frequency	21 (68)	12 (54)	9 (100)	0.1811
Lifestyle modifications	24 (77)	18 (81)	6 (67)	0.6385
Patient education	29 (94)	21 (95)	8 (89)	>0.99

*Statistical significance *p* < 0.01.

**Statistical significance *p* < 0.05.

### Aspirin prescribing practices

Most physicians (71%) reported that they would prescribe aspirin when a patient has one risk factor for EOP. All attending physicians and most residents/fellows (63%) fell into this category. However, residents/fellows (10%) also reported that they would prescribe prophylactic aspirin to all women, even if they had no risk factors for hypertension or preeclampsia, and 27% stated that patients must have more than one risk factor before they would recommend aspirin. Sixty-nine percent of physicians (residents/fellows and attendings) reported that the earliest they would recommend a woman take aspirin would be 9–13 weeks of gestation but recommendations to discontinue taking aspirin varied ranging from 20 weeks to birth (Table 3).

**Table 3.** Aspirin Prescribing Practices

	Total (***n*** = 31)	Resident/fellow (***n*** = 22)	Attending (***n*** = 9)	***p***
Lowest level of risk for aspirin prescription				
Patient must have >1 risk factor	6 (19)	6 (27)	0	0.2409
Patient must have 1 risk factor	22 (71)	14 (63)	9 (100)	
Patient has no risk factors	3 (10)	3 (10)	0	
Earliest point of prescribing aspirin in high-risk patients, *n* (%)^a^				
1–4 weeks	6 (21)	6 (26)	2 (25)	0.7548
5–8 weeks	1 (3)	1 (4)	0	
9–13 weeks	20 (69)	14 (61)	6 (75)	
14–17 weeks	2 (7)	2 (9)	0	
Point in time to discontinue aspirin, *n* (%)^b^				
By 20 weeks	2 (9)	1 (5)	1 (25)	0.6813
By 28 weeks	1 (4)	1 (5)	0	
By 34 weeks	5 (22)	4 (20)	1 (25)	
By 36 weeks	13 (56)	12 (60)	2 (50)	
When HTN is controlled	2 (9)	2 (10)	0	

^a^ *n* = 29 respondents.

^b^ *n* = 23 respondents.

### Barriers and facilitators to care

Residents/fellows were more likely to report barriers to prescribing aspirin than attendings and fellows. These barriers included limited evidence about aspirin as prevention of preeclampsia, patient compliance, lack of shared decision-making with the patient, and lack of knowledge about appropriate recommendations for different patient populations. Some attending physicians reported similar barriers, but they reported them with less frequency than residents/fellows.

Facilitators to prescribing aspirin were reported consistently by all physicians, regardless of training level. Facilitators were low cost of and easy access to aspirin, limited evidence of harm from taking aspirin during pregnancy, evidence of aspirin's effectiveness, and USPSTF recommendations. Attending physicians were more aware of the USPSTF recommendations than residents/fellows (78% vs. 54%) (Table 4).

**Table 4.** Barriers and Facilitators to Care

	Total (***n*** = 31)	Resident/fellow (***n*** = 22)	Attending (***n*** = 9)	***p***
Barriers to care, *n* (%)				
Limited evidence on prevention of preeclampsia	5 (16)	5 (26)	0	0.5295
Patient compliance	21 (68)	16 (72)	5 (56)	0.6039
Limited medication adherence	17 (55)	13 (59)	4 (44)	0.7268
Lack of shared decision-making	10 (32)	10 (45)	1 (11)	0.1541
Lack of knowledge of appropriate recommendations for different patient populations	17 (55)	12 (63)	4 (44)	0.9076
Facilitators to care, *n* (%)				
Cost of aspirin	30 (97)	21 (95)	8 (89)	>0.99
Ease of access to treatment	21 (68)	18 (82)	3 (33)	0.0303^*^
Limited evidence of significant harm from aspirin	30 (97)	21 (95)	9 (100)	>0.99
Evidence of aspirin's effectiveness	29 (94)	20 (90)	9 (100)	>0.99
USPSTF recommendations as endorsement	19 (61)	12 (54)	7 (78)	0.4291

^*^ Statistical significance *p* < 0.05.

USPSTF, United States Preventive Services Task Force.

## Discussion

Important differences between provider types in the prevention of hypertension and preeclampsia were observed. Resident physicians were less likely to discuss and provide educational materials about hypertension and preeclampsia to all patients compared to fellows and attendings. These differences by training level could be attributed to the lack of consistency between training guidelines and the lack of consistency between guidelines taught at medical schools or followed at training institutions.

Physicians (residents/fellows and attendings) in this study reported that shared decision-making with the patient was a barrier to providing preventive EOP care, which is consistent with a study conducted among pregnant Medicaid beneficiaries.^10^ This sentiment was more common among residents/fellows than attending physicians, which could be attributed to the lack of continuity of care in the resident clinics at our institution. A study published in 2021 found that the use of a smartphone app with data shared between patient and provider improved both the patient's and provider's ability to identify and manage risk factors for EOP.^10^ An intervention similar to this could be a strategy to increase shared decision-making between patient and provider, which could be especially impactful in clinics with low continuity of care.

Another reported barrier was a lack of patient compliance with aspirin use. Patients have reported barriers to aspirin use that include inadequate knowledge about the use of aspirin to prevent preeclampsia and its sequelae as well as perceived disagreements between providers regarding the use of prophylactic aspirin.^11^ These disputes could be attributed to the varying clinical guidelines. Regardless of the reason, these issues of patient noncompliance can be addressed by intervening at the health care level.

Physicians in this study reported access to care as a facilitator to prevention, treatment, and prescribing patterns, which is consistent with other studies found that access to care as a facilitator to prevention treatment and prescribing patterns.^12,13^

Our study also found that recommendations for care of high-risk patients varied by provider type. Particularly, resident physicians were more likely to refer all high-risk patients to high-risk obsetrcians compared to other provider types. Our study also found providers report limited clinical recommendations on treatment and prescribing patterns for certain populations. The finding is notable considering that Black race is a risk factor for preeclampsia. All results are consistent with a prior study that reported that MFM attendings were more likely to use certain practices, such as using a pre-eclampsia prediction algorithm and prescribing aspirin for chronic hypertensive patients and those with abnormal biomarkers such as vascular endothelial growth factor and placental growth factor.^14,15^

## Conclusions

Our study adds to the literature that suggests that regardless of provider type, providers are prepared to prevent and treat hypertension or preeclampsia, yet variation in recommendations, limited recommendations for different populations, and limited clinical trials present challenges. Therefore, additional clinical trials including different populations should be conducted to refine clinical recommendations.

## Abbreviations Used

ACOG: American College of Obstetricians and Gynecologists
EOP: Early onset preeclampsia
FIGO: International Federation of Gynecology and Obstetrics
LOP: late-onset preeclampsia
MFM: maternal fetal medicine
OBGYN: obstetric/gynecologic
USPSTF: United States Preventive Services Task Force
WHO: World Health Organization
